# Does the Installation or the Improvement of Existing Outdoor Parks Increase Physical Activity Levels? A Systematic Review

**DOI:** 10.3390/sports11110221

**Published:** 2023-11-09

**Authors:** Miguel Peralta, Gianluca Viscioni, Xavier Melo, Élvio R. Gouveia, Thorsten Griesser, Alexander Blocher, Maurizio Bertollo, Andrea Di Blasio, Adilson Marques

**Affiliations:** 1 Centro Interdisciplinar de Estudo da Performance Humana (CIPER), Faculdade de Motricidade Humana, Universidade de Lisboa, 1495-751 Cruz Quebrada, Portugal; amarques@fmh.ulisboa.pt; 2 Instituto de Saúde Ambiental (ISAMB), Faculdade de Medicina, Universidade de Lisboa, 1649-026 Lisbon, Portugal; 3 Department of Medicine and Aging Sciences, “G. d’Annunzio” University of Chieti-Pescara, 66100 Chieti, Italy; gianluca.viscioni@gmail.com (G.V.); m.bertollo@unich.it (M.B.); andrea.diblasio@unich.it (A.D.B.); 4 Centro de Investigação Interdisciplinar Egas Moniz (CiiEM), Escola Superior de Saúde Egas Moniz, 2829-511 Caparica, Portugal; xmelo@egasmoniz.edu.pt; 5 Department of Physical Education and Sport, University of Madeira, 9020-105 Funchal, Portugal; erubiog@uma.pt; 6 Laboratory of Robotics and Engineering Systems, Interactive Technologies Institute, 9020-105 Funchal, Portugal; 7 Planet O GmH, Post SV Nuremberg e.V., 90482 Nuremberg, Germany; griesser@planet-o.eu (T.G.); blocher@planet-o.eu (A.B.)

**Keywords:** blue exercise, fitness, green exercise, playground, public health

## Abstract

Investment in outdoor parks is proposed as a promising large-scale strategy to promote physical activity (PA). This study aimed to systematically review the impact of park renovations or installing new ones in increasing PA. Searches were conducted using predefined terms in three databases (PubMed, Scopus, and Web of Science) in March 2022. Studies examining the effectiveness of park renovations or developing new ones in increasing PA and having control or comparison were eligible for inclusion. Study quality was assessed using the Quality Assessment Tool for Quantitative Studies. Data were extracted from the included records using a predefined extraction table. The database search yielded 959 records, and 26 were included. For park renovations (n = 17), 11 (65%) studies presented findings supporting a positive effect on PA. The other six (35%) studies found no PA benefits when compared to control or pre-renovations/improvement levels. Regarding new installations (n = 9), five (56%) studies presented improvements in PA, and four (44%) did not. A promising positive impact of park renovations and new installations on park use and PA was observed. The review findings reflect the need to understand the context, daily routines, and interests of the surrounding population before renovating or installing new outdoor parks.

## 1. Introduction

Physical inactivity is the fourth leading cause of mortality, being responsible for approximately 3 million preventable deaths worldwide [1]. On the other hand, physical activity (PA) is associated with a lower risk of several disease outcomes, with the greatest gains occurring at lower activity levels [2]. Hence, the World Health Organization (WHO) formulated a global strategy to address PA and health from 2018–2030 [3], wherein a 15% relative reduction in the prevalence of insufficient PA was set as one of the goals.

Despite the extensive investment (i.e., a range of policies and initiatives implemented over the past decades to promote PA and reduce sedentary behaviour as public health priorities), the PA levels have remained relatively constant over the years [4]. Consequently, researchers and public health officials are exploring the role of large-scale strategies, such as improving access to PA with informational outreach activities, community-scale and street-scale urban design and land use, active transportation policy and practices, and community-wide policies and planning, as they all have previously led to acceptable increases in PA [5,6,7,8,9,10,11,12]. Health, well-being (e.g., stress reduction), social (e.g., crime reduction, improved perceptions of safety), and environmental (e.g., increased biodiversity) benefits have also been identified [13]. However, the causal effect of changes in the built environment on PA has an overall critical or serious risk of bias [14,15]. Still, developing a supportive environment has the potential to achieve [16] and maintain [17] the biggest reach for long-term, population-wide improvements in PA levels, with greater cost-effectiveness compared to individual-level interventions [18].

As these proposals are now starting to be reflected in policy guidelines for PA worldwide [19,20], it is essential to identify whether investments in renovations or building new infrastructures are equally important in initiating and helping to maintain PA behaviour. Although both renovating or constructing supportive environments can accrue multiple benefits to funders and residents, including improved property values [21], and a well-equipped, novel, and engaging place for recreation [22], these may differ on the feelings of ownership, responsibility, and sense of stewardship, improve perceptions of safety, and frequent use of the parks [23]. In addition, the capital costs of park renovations or construction may vary, as increasingly strict building codes now encompass more details, ranging from the materials used for park surfaces to the security standards for playground equipment, in addition to significant expenses for maintenance and repairs [22]. Therefore, this study aimed to undertake a systematic review to assess the impact of park renovations or installing new ones on the population/users’ PA levels.

## 2. Materials and Methods

This systematic review followed the Preferred Reporting Items for Systematic Reviews and Meta-analyses (PRISMA) 2020 checklist guidelines [24]. The systematic review is registered in PROSPERO (id: CRD42022319863).

### 2.1. Search Strategy

The population, interventions, comparisons, outcomes, and study design (PICOS) framework was used to guide the research question formulation and systematic search [25], which resulted in the following terms: (fitness OR “physical activit*” OR active OR sport* OR exercise* OR play OR activity) AND (outdoor* OR nature OR lake OR river OR greenspace* OR “green space*” OR greenway OR park OR parks OR playground* OR spot OR spots OR space OR spaces OR “blue environment*” OR “blue space” OR “beach*” OR “seaside”) AND (new OR change OR changing OR renovati* OR built OR installation OR intervention* OR renew OR renewal OR improve OR improvement*). Following this formulation, on 31 March 2022, the search was conducted on the PubMed, Scopus, and Web of Science databases. Afterward, the reference section of the included studies was searched for additional records.

### 2.2. Study Selection

Following the initial identification through a database search, all records were retrieved and organised using reference management software (EndNote 20, Clarivate, Philadelphia, PA, USA). Duplicate entries were removed. Then, two authors (GV; MP) screened the title and abstract of the records and excluded those deemed outside of the scope. Lastly, two authors (MP; AM) assessed full-text records for eligibility guided by the inclusion criteria. All disagreements were solved by consensus.

For studies to be included in the review, the following eligibility criteria were applied: children, adolescents, adults, or older adults (population criteria); examining a new outdoor park/playground or improvement of existing outdoor park/playground (intervention/exposure criteria); comparing with previous PA or having a control group (comparison criteria); having results on PA levels (outcome criteria); observational and experimental studies (study type criteria); and published in English, French, Italian, Portuguese or Spanish (language criteria).

### 2.3. Data Extraction

Records included in the systematic review were incorporated into the data extraction process, carried out by two authors, MP and AM. To extract data, a spreadsheet was generated with the following fields as columns: authorship, publication year, study design, sample, country, intervention/exposure, control/comparison, outcomes, instruments, and main findings. Both authors completed the spreadsheet by inputting the information for each article, and the two authors deliberated upon the extracted data to create the final data extraction spreadsheet. This spreadsheet was subsequently utilised to create the results tables.

### 2.4. Study Quality and Risk of Bias

The Quality Assessment Tool for Quantitative Studies [26] was used to assess the study quality. This tool comprises a set of questions allocated to specific sections, including selection bias, study design, confounders, blinding, data collection methods, withdrawals, and dropouts. Each section was graded as weak, moderate, or strong according to the specified criteria. Ultimately, a global rating is determined, given the scores of each section. Two authors assessed study quality independently (MP, AM), and discrepancies were discussed and resolved by consensus.

## 3. Results

### 3.1. Literature Search

**Figure 1:**
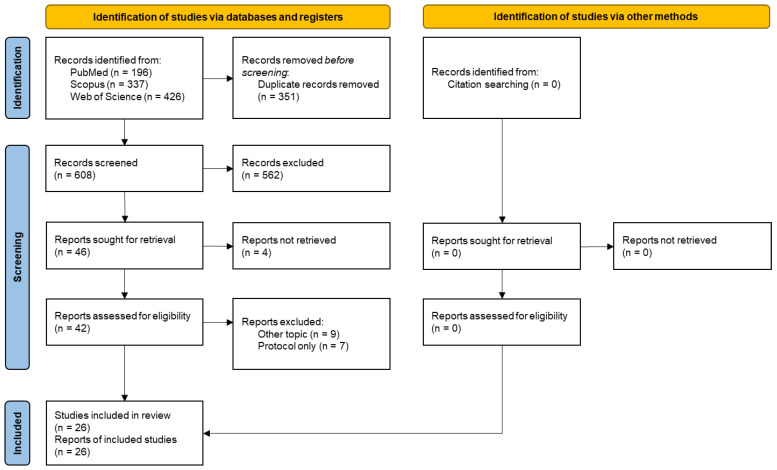
Flowchart of the study selection process.

The flowchart of the study selection process is presented in Figure 1. Database search yielded 959 records (196 from PubMed, 337 from Scopus, and 426 from Web of Science), and 351 duplicates were eliminated. The remaining 608 records entered the title and abstract screening stage, of which 562 were removed. A total of 46 records were sought for retrieval, and 4 were not accessible (only the abstract version was found). In the eligibility stage, 42 records were analysed, and 16 were excluded (9 were focused on other topics, and 7 were only protocols). The citation search identified no records. This resulted in 26 records being included in the systematic review.

### 3.2. Study Characteristics

Of the 26 included articles, 15 had an observational design, 8 had an intervention design, and 3 had a quasi-experimental design. The studies included participants from several countries, including fourteen from the United States, four from Australia, two from Denmark, and one from the following countries England, Chile, China, the Netherlands, Northern Ireland, and Thailand. Presented outcomes were park use, PA, and sedentary behaviour. The instruments used to measure PA were the System for Observing Play and Recreation in Communities (SOPARC) in 15 studies, accelerometers in 3 studies, own questionnaire in 3 studies (two self-reports, one parent report), System of Observing Play and Leisure Activity in Youth (SOPLAY) in 2 studies, and the Global Physical Activity Questionnaire (GPAQ), the International Physical Activity Questionnaire (IPAQ), and pedometers in 1 study each.

### 3.3. Main Findings

To facilitate comprehension, the included studies were divided into two groups: (a) 17 studies assessing renovations or improvements to existing infrastructures (e.g., renovating a park or installing fitness equipment on an already existing park); and (b) 9 studies assessing the availability of new infrastructure (e.g., developing a new greenway).

Table 1 presents the characteristics and main findings for studies assessing renovations or improvements to existing infrastructures. Of the 17 studies, 11 (65%) presented findings supporting a positive effect of park renovations or improvements in the population/users’ PA levels [21,22,27,28,29,30,31,32,33,34,35]. On the other hand, the remaining six (35%) studies found no benefits in PA compared to control or pre-renovations/improvement levels [23,36,37,38,39,40]. Also, among the 17 studies on renovations, 12 were focused on major renovations in public parks (8/12 [67%] showed positive findings), 4 on renovations to school playgrounds (3/4 [75%] showed positive findings), and 1 on extending an existing greenway (showed no effect).

The characteristics and main findings of the studies assessing the availability of new infrastructure are presented in Table 2. Overall, of the nine studies in this group, five (56%) presented benefits on PA levels [41,42,43,44,45], while the other four (44%) did not [46,47,48,49]. Furthermore, four studies were focused on developing new greenways (1/4 [25%] showed positive findings), three were focused on building new recreational spaces for PA (2/3 [67%] showed positive findings), and two were focused on making available previously closed spaces dedicated to PA (2/2 [100%] showed positive findings).

Some characteristics of the included studies, such as outcome, instrument used, control/comparator conditions, and age, were also isolated to provide a more detailed view of the findings, independent of renovations or new installments. The main outcome assessed was PA, with park use and sedentary behaviour as secondary outcomes. Of the included studies, little more than half (16/26 [62%]) showed a small to medium improvement in PA levels. On the other hand, most studies showed improvements in park use (12/14 [86%]). Only three studies had sedentary behaviour as an outcome, and two showed reductions after the intervention (2/3 [67%]). Objective and subjective instruments and direct observation were used to assess outcomes. When using self-report instruments (e.g., questionnaires), the effectiveness prevalence was the lowest (1/5 [20%]). Contrary to this, when using objective instruments (accelerometers or pedometers) the effectiveness prevalence was the highest (3/4 [75%]). The most used instrument type was direct observation, using SOPARC and SOPLAY, and results revealed that most interventions successfully increased PA and/or park use (12/17 [71%]).

As for participants’ characteristics, only one group could be isolated for analysis: children and adolescents. Eight studies have been conducted on young people, from kindergarten to high school, and a little more than half were effective in increasing PA (5/8 [63%]).

Different control/comparator conditions were used to assess the effect of the renovations or new constructions. These conditions can be grouped into three: comparison with the same location before the intervention, controlling with other similar locations, and comparing with both other locations and the same location before. Comparing the same place before and after the intervention yielded the biggest success rate (6/8 [75%]), followed by the studies controlling for other locations (7/12 [58%]). Six studies had both comparisons, and only two (2/6 [33%]) found the intervention effective in improving the outcome.

### 3.4. Study Quality and Risk of Bias

Table 3 summarises the study quality assessment using the Quality Assessment Tool for Quantitative Studies. Overall, 10 studies (38%) presented a weak methodological quality, 13 studies (50%) had a moderate methodological quality, and 3 studies (12%) had strong methodological quality.

## 4. Discussion

This systematic review aimed to assess the impact of park renovations and the installation of new outdoor parks on the PA levels of the population/users. The results showed a promising positive impact of park renovation and new installations on park use and PA. More studies were focused on renovations than on new installations, and a greater percentage of studies focused on renovations than new installations (65% vs. 56%) showed a positive impact on PA. However, the number of studies is quite low and mainly represents English-speaking countries.

Previous studies have underlined the necessity for a successful intervention to combine built-environment changes in parks with programming to increase awareness of the park and park use, according to local constraints [15,39]. Although 6 of the 17 studies focused on park renovation did not find direct PA benefits, 3 still showed increased park use. Two of those had interventions at building park awareness [23,39]. Many other included studies have also reported increased park use isolated and increased PA. While increases in PA show that users are more active, increases in park use may reflect reaching new users and more frequent park use, potentially leading to more active users. Thus, increasing park use is also an important outcome. Notwithstanding, little is known about the effect of tailored interventions on the translation of park use to improved PA levels. Future research should try to understand the impact of programming on the effectiveness of built environment interventions. This may be a key factor in the decision-making process and the effective investment outcome.

The determinants of PA throughout the life course are complex, spanning from policy [50], biological [51], socio-cultural [52], socio-economic [53], psychological [54] and behavioural [55] domains. This means that to properly implement an effective intervention and/or to reproduce a successful experience, it is necessary to know whether all the necessary prerequisites are met, especially when investments from public funds are present. Therefore, park renovations or constrictions to increase park use should be part of a more comprehensive and coordinated project examining the determinants, correlates, and mediators of PA. For example, school playground renovations positively impacted young people’s PA [27,28,31,38]. One study showed no impact among Danish adolescents [38], while the others were conducted among children. On the other hand, developing new greenways and expanding existing greenways was mostly ineffective in increasing PA levels [36,46,48]. Smaller-scale interventions (school playground vs. public park) and a stricter target audience (school-aged children vs. all park users/community) may explain the greater success of school playground interventions. This reflects the importance of understanding the context, daily routines, and interests of the surrounding population before renovating or installing new outdoor parks.

Interestingly, the availability of previously closed spaces dedicated to PA was revealed to be an effective strategy for increasing PA. However, only two studies investigated this situation. This strategy was used to open a schoolyard after school hours for children to play [41] and close four continuous blocks to traffic for three hours twice a week [44]. Strategies like these optimise using existing resources, which are familiar to the surrounding communities [56]. Also, it allows for adaptability, targeted approaches, and investing the saved funds in upgrading or dynamising those spaces. However, more studies are needed to confirm the efficacy of this strategy and to which extent they are a better alternative to the new construction of outdoor parks.

The included studies were considerably heterogeneous regarding the methods employed, including outcome assessment, control condition, and participant characteristics. However, analysing possible factors associated with the effectiveness of the interventions (renovations or new installments) is important to increase the application of the findings. More studies showed improved park use (86%) than PA levels (62%). While increasing park utilisation is a positive step, as it may indicate the engagement of new users and more frequent visits from previous users, further actions are required to enhance physical activity beyond mere infrastructural interventions. For example, a systematic review assessing infrastructural interventions to improve cycling behaviour found that both use and behaviour intention were important to consider when evaluating these interventions [57].

Analysis isolating the instrument used to assess the outcome revealed that studies using objective measurements (accelerometers and pedometers) had a greater prevalence of effectiveness than studies using self-reported measurements. This was unexpected as generally self-reported measures of PA are greater than objective measures [58], but may be related to the characteristics of the interventions as most of these studies were focused on playgrounds and thus tailored to a specific target audience (children). Notwithstanding, subjective measures can also underrepresent people’s PA levels [59]. Future studies must attempt to use objective measures more often to improve data quality on this topic.

Another important indication to consider is the type of control/comparison. Studies comparing the same location to a previous point in time (mostly renovations) were more effective than those controlling/comparing to similar locations (e.g., parks, playgrounds). This can be related to previous knowledge and awareness of the park’s existence [56] and more direct comparisons between sites. Furthermore, this study’s findings showed that studies focused on renovations seemed more effective than those focused on new installations, supporting the idea that having a previous connection to the intervened facility may be beneficial.

There are a set of limitations that should be considered when interpreting the findings of this systematic review. First, the included studies varied in design, population, and data collection methodology. This makes comparison difficult and induces heterogeneity in the findings. Also, most studies provided little information on participants’ characteristics, which does not allow full interpretation of the observed results. Secondly, the review findings can only be contextualised in the territories/neighbourhoods/schools where the studies were conducted. Even in the same country, the geographical characteristics of a territory can promote or hinder the success of the same intervention. Thus, care should be taken when generalising the findings. Thirdly, although study quality was assessed, studies were not weighted or ranked, nor were any removed from the review. Therefore, studies with weaker quality were not given less importance than findings from studies with higher quality. Lastly, other potential sources of information, such as websites, were not considered.

## 5. Conclusions

A promising positive impact of both park renovation and new installations on park use and PA was observed, with a higher percentage of studies focused on renovations than on new installations (65% vs. 56%) showing a positive impact on PA. The review of the existing literature provides evidence of the importance of a tailored approach, not just to create new parks or renovate existing ones but to provide adequate parks according to the context and population characteristics, together with fostering awareness. This reflects the need to understand the context, daily routines, and interests of the surrounding population before renovating or installing new outdoor parks.

## Figures and Tables

**Table 1:** Characteristics and main findings of studies assessing renovations or improvements to existing infrastructures.

Author(s), Year	Study Design; Sample (% Male); Age	Country	Intervention/Action	Control/ Comparison	Outcome (s)	Instrument (s)	Main Findings
Hannon & Brown, 2008 [27]	Intervention; n = 64 (46.8%); Age range: 3–5 years	USA	Play equipment added to a preschool playground.	Previous PA.	PA and SB	Accelerometers	The time spent in sedentary behaviour decreased by 16%. Light, moderate, and vigorous PA increased by 3.5%, 7.8%, and 4.5%, respectively.
Cohen, et al., 2009 [23]	Observational; Baseline n = 1535, Follow-up n = 1332; Median age range: 36.5–40.5 years	USA	Selected parks had major renovations.	Similar parks (not renovated).	PU and PA	SOPARC	Overall, PU and PA decreased from baseline to follow-up in both the intervention and control parks. However, compared to the control, intervention parks attracted more new users (50% vs. 25%).
Tester & Baker, 2009 [21]	Observational; Baseline n= 1006 (84.4%), Follow-up n= 3883 (73.0%); Children to older adults	USA	2 parks had major renovations.	Similar park (not renovated).	PU and PA	SOPARC	An increase in playground users was observed in the renewed parks but not in the control. Visitors’ MPA and VPA increased by 3 and 2 times in the renewed parks (compared to the baseline).
Ridgers, Fairclough, & Stratton, 2010 [28]	Intervention; n = 470 (49.3%); Elementary and primary school-aged children	England	15 schools redesigned their playground.	11 matched control schools.	PA	Accelerometers and heart rate monitors	Intervention children participated in 4% more VPA than the control. The effect of the intervention was significant and positive at 6 months after the intervention for MVPA and VPA but reduced at 12 months.
West & Shores, 2011 [36]	Observational; n = 169 (47.6%); Adults and older adults	USA	Adding a 5-mile greenway to an existing greenway along a river.	Previous PA and people living further away from the greenway.	PA	Questionnaire	Small but non-significant increases in walking, moderate, and vigorous activity were observed. The distance from the house to the greenway did not present significant interactions.
Veitch et al., 2012 [29]	Observational; n = 2050 (53.5%); Children to older adults	Australia	Public park that had renovations.	Control park (not renovated).	PU and PA	SOPARC	Significant increases in the number of park users, people walking, and vigorously active people post renovations.
Bohn-Goldbaum et al., 2013 [37]	Quasi-experimental; n = 140; Children (age range: 2–12 years) and their parents	Australia	Park equipment and green space added to a public park.	Control park (not renovated).	PU and PA	SOPARC	No differences between the intervention and control parks were found in PU and MVPA. Significant decrease in girls’ MVPA levels in the renovated playground.
Toftager et al., 2014 [38]	Intervention; n = 797 (49.4%); Mean age: 12.5 years	Denmark	Improvements to the environment (e.g., playground) of 7 schools.	7 control schools.	PA	Accelerometers	No evidence was found of the overall effect of the intervention on PA.
Cohen et al., 2015 [22]	Observational; n = 924 (55.5%); Mean age: 43 years	USA	2 parks that had renovations.	4 control parks (2 not renovated; 2 partly renovated).	PU and PA	SOPARC	PU and PA increased in the renovated parks and decreased in the parks that were not renovated.
Slater et al., 2016 [30]	Quasi-experimental; n = 78 parks; No age information	USA	39 parks that had renovations.	39 control parks (not renovated).	PU and PA	SOPARC	Significant increases were found in PU (6.51%) and the number of people participating in MVPA (7.88%)
Frost et al., 2018 [31]	Intervention; n = 148; 5th and 6th graders	USA	Playground redesigned.	Same playground before redesign.	PA	SOPLAY	The percentage of children engaging in MVPA and VPA increased by 23.3% and 26.2% at 6-month follow-up. These increases were sustained at 1-year follow-up.
Sami, Smith, & Ogunseitan, 2018 [32]	Intervention; Pre-intervention n = 1650 person-period, Post intervention n = 1776 person-period; No age information	USA	Fitness equipment installed in a public park.	Same park before installation.	PU and PA	SOPARC	Post-intervention users had 58% and 41% higher odds for a higher activity level than pre-intervention users in the new fitness area and the whole park, respectively.
Veitch et al., 2018 [33]	Observational; n = 15,305 (49.4%); Children to older adults	Australia	Installation of a playscape in a large metropolitan park	Control park (not renovated) and same park before renovations.	PU and PA	SOPARC	Increase in PU (176%) and users were engaging in MVPA (119%) at 12-month follow-up compared to the control park.
Cohen et al., 2019 [34]	Observational; n = 2570; Children to older adults	USA	5 parks that had renovations.	Control park (not renovated) and same parks before renovations.	PU and PA	SOPARC	The renovated parks showed increases in PA, while the control park showed a 45% decrease in PA (MET hours per observation).
Arifwidodo & Chandrasiri, 2021 [35]	Observational; Baseline n = 11,309, Follow-up n = 12,504; Children to older adults	Thailand	Park that had renovations.	Same park before renovations.	PU and PA	SOPARC	Increases in PU (4.1%) and the number of users cycling and running after renovations (17.6%).
Kelly, Clennin & Hughey, 2021 [39]	Observational; Baseline n = 144, Follow-up n = 219; No age information.	USA	2 parks that had renovations.	Same parks before renovations.	PU and PA	SOPARC	In one of the parks, the PU increased by 53%. Changes in PA were not significant in both parks.
Veitch et al., 2021 [40]	Intervention; Baseline n = 1514 (60.8%), Follow-up n = 1907; Children to older adults	Australia	Park that had renovations.	Control park (not renovated) and same park before renovations.	PU and PA	SOPARC	No significant changes were observed in PU and PA.

Abbreviations: MPA, moderate physical activity; MVPA: moderate-to-vigorous physical activity; PA, physical activity; PU, park usage; SOPARC, System for Ob-serving Play and Recreation in Communities; SOPLAY, System for Observing Play and Leisure Activity in Youth; USA, United States of America; VPA, vigorous physical activity.

**Table 2:** Characteristics and main findings of studies assessing the availability of new infrastructure.

Author(s), Year	Study Design; Sample (% Male); Age	Country	Intervention/Action	Control/Comparison	Outcome (s)	Instrument (s)	Main Findings
Farley et al., 2007 [41]	Intervention; n = 1465; Preschool to 6th graders	USA	The schoolyard was open after school dismissal for the children to play.	Control neighbourhood (schoolyard remained locked).	PA and SB	SOPLAY modified version	30% more active children in the intervention neighbourhood. Screen time decreased in the intervention neighbourhood and increased in the comparison neighbourhood.
King et al., 2015 [42]	Observational; n = 7413 (55.2%); Children to older adults	USA	Undeveloped green space transformed into a recreational park.	Same and adjacent locations before availability of new park.	PU and PA	SOPARC	Park location presented a 3-fold increase in PA (energy expended within the park).The % of adolescent males observed in VPA increased by 27%.
Schipperijn, Hansen & Rask, 2015 [43]	Observational; n = 331, (70.3%); Children to older adults	Denmark	Installation of 3 bicycle playgrounds.	No control or comparison.	PU and PA	SOPARC and interviews	63% of the users were active.
West & Shores, 2015 [49]	Observational; n = 273 (57.5%); Children to older adults	USA	A new greenway/trail was built.	Control neighbourhood (located 2–3 miles of the greenway)	PA	Questionnaire	No differences were found in walking, MPA, and VPA before and after the constructed greenway. The construction of a greenway did not affect the PA of the proximate residents.
Cortinez-O’Ryan et al., 2017 [44]	Intervention; Intervention n = 59 (53%), Control n = 49 (45%); Median age range: 7–9 years	Chile	4 continuous blocks were closed to traffic for 3 h twice a week for 4 months.	Control neighbourhood.	PA	Pedometers	Increases in daily steps and outdoor playtime after school were observed in the intervention group. No changes were observed in the control group. The % of children who met the recommended daily steps increased by 25, 5% in the intervention neighbourhood.
Auchincloss et al., 2019 [46]	Quasi-experimental; n = 8783 observations; No age information.	USA	Construction of a 1.5-mile greenway.	Control streets and same location before greenway.	PA	SOPARC	Small increases in MVPA (2%) after the greenway construction. However, the same increases were found in the control area.
Mölenberg et al., 2019 [47]	Observational; Exposed n = 171 (43.9%), Control n = 1670 (50.8%); Mean age range: 6.0–9.7 years	the Netherlands	Development of 13 new PA spaces within 600m from home.	Control group (children not exposed to new PA spaces).	PA and SB	Parent report	The development of PA spaces did not affect outdoor play or SB compared with the control. However, it may increase the time spent playing outdoors for children from socioeconomically disadvantaged families.
Hunter et al., 2021 [48]	Observational; Baseline n = 1037 (41%), Follow-up n = 968 (44.5%); Mean age range: 50.3–51.7 years	Northern Ireland	Development of a 9 km urban greenway.	Control area and same location before greenway.	PA	GPAQ	A slight reduction in PA levels after the development of the greenway was observed (68% to 61%).
Xie et al., 2021 [45]	Observational; n = 1020 (43.4%); Mean age: 50.8 years	China	Development of a 102 km urban greenway.	Same location before the greenway.	PA	IPAQ	At follow-up, MVPA and overall PA increased by 9.5% and 10.4% compared to baseline. In addition, PA benefits decrease with increasing distance between the greenway and the residence.

Abbreviations: QPAQ, Global Physical Activity Questionnaire; IPAQ, International Physical Activity Questionnaire; MPA, moderate physical activity; MVPA: moderate-to-vigorous physical activity; PA, physical activity; PU, park usage; SB, sedentary behaviour; SOPARC, System for Observing Play and Recreation in Communities; SOPLAY, System for Observing Play and Leisure Activity in Youth; USA, United States of America; VPA, vigorous physical activity.

**Table 3:** Summary of study quality using the Quality Assessment Tool for Quantitative Studies.

Study	Section						Global Rating
	A	B	C	D	E	F	
Farley et al., 2007 [41]	Moderate	Moderate	Strong	Weak	Strong	Strong	Moderate
Hannon & Brown, 2008 [27]	Moderate	Moderate	Strong	Weak	Strong	Strong	Moderate
Cohen, et al., 2009 [23]	Moderate	Moderate	Strong	Weak	Strong	Strong	Moderate
Tester & Baker, 2009 [21]	Moderate	Moderate	Weak	Moderate	Strong	Strong	Moderate
Ridgers, Fairclough, & Stratton, 2010 [28]	Strong	Moderate	Weak	Moderate	Strong	NA	Moderate
West & Shores, 2011 [36]	Moderate	Moderate	Weak	Moderate	Strong	NA	Moderate
Veitch et al., 2012 [29]	Moderate	Moderate	Weak	Moderate	Strong	NA	Moderate
Bohn-Goldbaum et al., 2013 [37]	Moderate	Moderate	Strong	Weak	Strong	Weak	Weak
Toftager et al., 2014 [38]	Strong	Strong	Strong	Weak	Strong	Strong	Moderate
Cohen et al., 2015 [22]	Moderate	Moderate	Strong	Weak	Strong	NA	Moderate
King et al., 2015 [42]	Moderate	Moderate	Weak	Weak	Strong	NA	Weak
Schipperijn, Hansen & Rask, 2015 [43]	Moderate	Moderate	Weak	Weak	Strong	NA	Weak
West & Shores, 2015 [49]	Weak	Moderate	Strong	Weak	Weak	Weak	Weak
Slater et al., 2016 [30]	Moderate	Moderate	Strong	Weak	Strong	Weak	Weak
Cortinez-O’Ryan et al., 2017 [44]	Moderate	Moderate	Strong	Weak	Strong	NA	Moderate
Frost et al., 2018 [31]	Moderate	Moderate	Strong	Weak	Strong	Strong	Moderate
Sami, Smith, & Ogunseitan, 2018 [32]	Moderate	Moderate	Weak	Moderate	Strong	Weak	Weak
Veitch et al., 2018 [33]	Moderate	Moderate	Strong	Moderate	Strong	Weak	Moderate
Auchincloss et al., 2019 [46]	Moderate	Moderate	Strong	Moderate	Strong	NA	Strong
Cohen et al., 2019 [34]	Moderate	Moderate	Strong	Weak	Strong	Weak	Weak
Mölenberg et al., 2019 [47]	Moderate	Moderate	Strong	Weak	Weak	Strong	Weak
Arifwidodo & Chandrasiri, 2021 [35]	Moderate	Moderate	Strong	Moderate	Strong	NA	Strong
Hunter et al., 2021 [48]	Weak	Moderate	Strong	Weak	Strong	Weak	Weak
Kelly, Clennin & Hughey, 2021 [39]	Moderate	Moderate	Strong	Weak	Strong	NA	Moderate
Veitch et al., 2021 [40]	Moderate	Moderate	Strong	Moderate	Strong	NA	Strong
Xie et al., 2021 [45]	Weak	Moderate	Strong	Weak	Strong	Weak	Weak

Sections: A, selection bias; B, design; C, confounders; D, blinding; E, data collection methods; F, withdraws and dropouts. Abbreviations: NA, not applicable.

## Data Availability

No new data were created or analysed in this study. Data sharing is not applicable to this article.

## References

[1] WHO (2012). Report of the Formal Meeting of Member States to Conclude the Work on the Comprehensive Global Monitoring Framework, including Indicators, and a Set of Voluntary Global Targets for the Prevention and Control of Noncommunicable Diseases.

[2] Kyu H.H., Bachman V.F., Alexander L.T., Mumford J.E., Afshin A., Estep K., Veerman J.L., Delwiche K., Iannarone M.L., Moyer M.L. (2016). Physical activity and risk of breast cancer, colon cancer, diabetes, ischemic heart disease, and ischemic stroke events: Systematic review and dose-response meta-analysis for the Global Burden of Disease Study 2013. BMJ.

[3] WHO (2018). Global Action Plan on Physical Activity 2018–2030: More Active People for a Healthier World.

[4] Guthold R., Stevens G.A., Riley L.M., Bull F.C. (2018). Worldwide trends in insufficient physical activity from 2001 to 2016: A pooled analysis of 358 population-based surveys with 19 million participants. Lancet Glob. Health.

[5] Heath G.W., Brownson R.C., Kruger J., Miles R., Powell K.E., Ramsey L.T., Task Force on Community Preventive Services (2006). The Effectiveness of Urban Design and Land Use and Transport Policies and Practices to Increase Physical Activity: A Systematic Review. J. Phys. Act. Health.

[6] Brownson R.C., Haire-Joshu D., Luke D.A. (2006). Shaping the context of health: A review of environmental and policy approaches in the prevention of chronic diseases. Annu. Rev. Public. Health.

[7] Heath G.W., Parra D.C., Sarmiento O.L., Andersen L.B., Owen N., Goenka S., Montes F., Brownson R.C., Lancet Physical Activity Series Working Group (2012). Evidence-based intervention in physical activity: Lessons from around the world. Lancet.

[8] Pucher J., Dill J., Handy S. (2010). Infrastructure, programs, and policies to increase bicycling: An international review. Prev. Med..

[9] Saelens B.E., Handy S.L. (2008). Built environment correlates of walking: A review. Med. Sci. Sports Exerc..

[10] MacMillan F., George E.S., Feng X., Merom D., Bennie A., Cook A., Sanders T., Dwyer G., Pang B., Guagliano J.M. (2018). Do Natural Experiments of Changes in Neighborhood Built Environment Impact Physical Activity and Diet? A Systematic Review. Int. J. Env. Res. Public Health.

[11] Smith M., Hosking J., Woodward A., Witten K., MacMillan A., Field A., Baas P., Mackie H. (2017). Systematic literature review of built environment effects on physical activity and active transport—An update and new findings on health equity. Int. J. Behav. Nutr. Phys. Act..

[12] Ferdinand A.O., Sen B., Rahurkar S., Engler S., Menachemi N. (2012). The relationship between built environments and physical activity: A systematic review. Am. J. Public Health.

[13] Hunter R.F., Cleland C., Cleary A., Droomers M., Wheeler B.W., Sinnett D., Nieuwenhuijsen M.J., Braubach M. (2019). Environmental, health, wellbeing, social and equity effects of urban green space interventions: A meta-narrative evidence synthesis. Environ. Int..

[14] Benton J.S., Anderson J., Hunter R.F., French D.P. (2016). The effect of changing the built environment on physical activity: A quantitative review of the risk of bias in natural experiments. Int. J. Behav. Nutr. Phys. Act..

[15] Hunter R.F., Christian H., Veitch J., Astell-Burt T., Hipp J.A., Schipperijn J. (2015). The impact of interventions to promote physical activity in urban green space: A systematic review and recommendations for future research. Soc. Sci. Med..

[16] Marteau T.M., Ogilvie D., Roland M., Suhrcke M., Kelly M.P. (2011). Judging nudging: Can nudging improve population health?. BMJ.

[17] Kwasnicka D., Dombrowski S.U., White M., Sniehotta F. (2016). Theoretical explanations for maintenance of behaviour change: A systematic review of behaviour theories. Health Psychol. Rev..

[18] Wu S., Cohen D., Shi Y., Pearson M., Sturm R. (2011). Economic analysis of physical activity interventions. Am. J. Prev. Med..

[19] Lee K.K. (2012). Developing and implementing the Active Design Guidelines in New York City. Health Place.

[20] Agence Nationale de la Cohésion des Territoires (2021). Guide du Design Actif.

[21] Tester J., Baker R. (2009). Making the playfields even: Evaluating the impact of an environmental intervention on park use and physical activity. Prev. Med..

[22] Cohen D.A., Han B., Isacoff J., Shulaker B., Williamson S., Marsh T., McKenzie T.L., Weir M., Bhatia R. (2015). Impact of park renovations on park use and park-based physical activity. J. Phys. Act. Health.

[23] Cohen D.A., Golinelli D., Williamson S., Sehgal A., Marsh T., McKenzie T.L. (2009). Effects of park improvements on park use and physical activity: Policy and programming implications. Am. J. Prev. Med..

[24] Page M.J., McKenzie J.E., Bossuyt P.M., Boutron I., Hoffmann T.C., Mulrow C.D., Shamseer L., Tetzlaff J.M., Akl E.A., Brennan S.E. (2021). The PRISMA 2020 statement: An updated guideline for reporting systematic reviews. BMJ.

[25] Schardt C., Adams M.B., Owens T., Keitz S., Fontelo P. (2007). Utilization of the PICO framework to improve searching PubMed for clinical questions. BMC Med. Inf. Decis. Mak..

[26] Thomas B.H., Ciliska D., Dobbins M., Micucci S. (2004). A process for systematically reviewing the literature: Providing the research evidence for public health nursing interventions. Worldviews Evid.-Based Nurs..

[27] Hannon J.C., Brown B.B. (2008). Increasing preschoolers’ physical activity intensities: An activity-friendly preschool playground intervention. Prev. Med..

[28] Ridgers N.D., Fairclough S.J., Stratton G. (2010). Twelve-month effects of a playground intervention on children’s morning and lunchtime recess physical activity levels. J. Phys. Act. Health.

[29] Veitch J., Ball K., Crawford D., Abbott G.R., Salmon J. (2012). Park improvements and park activity: A natural experiment. Am. J. Prev. Med..

[30] Slater S., Pugach O., Lin W.T., Bontu A. (2016). If You Build It Will They Come? Does Involving Community Groups in Playground Renovations Affect Park Utilization and Physical Activity?. Environ. Behav..

[31] Frost M.C., Kuo E.S., Harner L.T., Landau K.R., Baldassar K. (2018). Increase in Physical Activity Sustained 1 Year After Playground Intervention. Am. J. Prev. Med..

[32] Sami M., Smith M., Ogunseitan O.A. (2018). Changes in Physical Activity After Installation of a Fitness Zone in a Community Park. Prev. Chronic Dis..

[33] Veitch J., Salmon J., Crawford D., Abbott G., Giles-Corti B., Carver A., Timperio A. (2018). The REVAMP natural experiment study: The impact of a play-scape installation on park visitation and park-based physical activity. Int. J. Behav. Nutr. Phys. Act..

[34] Cohen D.A., Han B., Isacoff J., Shulaker B., Williamson S. (2019). Renovations of neighbourhood parks: Long-term outcomes on physical activity. J. Epidemiol. Community Health.

[35] Arifwidodo S.D., Chandrasiri O. (2021). The effects of park improvement on park use and park-based physical activity. J. Archit. Urban..

[36] West S.T., Shores K.A. (2011). The Impacts of Building a Greenway on Proximate Residents’ Physical Activity. J. Phys. Act. Health.

[37] Bohn-Goldbaum E.E., Phongsavan P., Merom D., Rogers K., Kamalesh V., Bauman A.E. (2013). Does playground improvement increase physical activity among children? A quasi-experimental study of a natural experiment. J. Environ. Public Health.

[38] Toftager M., Christiansen L.B., Ersboll A.K., Kristensen P.L., Due P., Troelsen J. (2014). Intervention Effects on Adolescent Physical Activity in the Multicomponent SPACE Study: A Cluster Randomized Controlled Trial. PLoS ONE.

[39] Kelly C., Clennin M., Hughey M. (2021). A Natural Experiment: Results of Community-Designed Park Improvements on Park Use and Physical Activity. Health Promot. Pract..

[40] Veitch J., Salmon J., Abbott G., Timperio A., Sahlqvist S. (2021). Understanding the impact of the installation of outdoor fitness equipment and a multi-sports court on park visitation and park-based physical activity: A natural experiment. Health Place.

[41] Farley T.A., Meriwether R.A., Baker E.T., Watkins L.T., Johnson C.C., Webber L.S. (2007). Safe play spaces to promote physical activity in inner-city children: Results from a pilot study of an environmental intervention. Am. J. Public Health.

[42] King D.K., Litt J., Hale J., Burniece K.M., Ross C. (2015). ‘The park a tree built’: Evaluating how a park development project impacted where people play. Urban. For. Urban. Green..

[43] Schipperijn J., Hansen C.K., Rask S. (2015). Use and activity levels on newly built bicycle playgrounds. Urban. For. Urban. Green..

[44] Cortinez-O’Ryan A., Albagli A., Sadarangani K.P., Aguilar-Farias N. (2017). Reclaiming streets for outdoor play: A process and impact evaluation of “Juega en tu Barrio” (Play in your Neighborhood), an intervention to increase physical activity and opportunities for play. PLoS ONE.

[45] Xie B., Lu Y., Wu L., An Z. (2021). Dose-response effect of a large-scale greenway intervention on physical activities: The first natural experimental study in China. Health Place.

[46] Auchincloss A.H., Michael Y.L., Kuder J.F., Shi J., Khan S., Ballester L.S. (2019). Changes in physical activity after building a greenway in a disadvantaged urban community: A natural experiment. Prev. Med. Rep..

[47] Molenberg F.J.M., Noordzij J.M., Burdorf A., van Lenthe F.J. (2019). New physical activity spaces in deprived neighborhoods: Does it change outdoor play and sedentary behavior? A natural experiment. Health Place.

[48] Hunter R.F., Adlakha D., Cardwell C., Cupples M.E., Donnelly M., Ellis G., Gough A., Hutchinson G., Kearney T., Longo A. (2021). Investigating the physical activity, health, wellbeing, social and environmental effects of a new urban greenway: A natural experiment (the PARC study). Int. J. Behav. Nutr. Phys. Act..

[49] West S.T., Shores K.A. (2015). Does Building a Greenway Promote Physical Activity Among Proximate Residents?. J. Phys. Act. Health.

[50] Puggina A., Aleksovska K., Buck C., Burns C., Cardon G., Carlin A., Chantal S., Ciarapica D., Condello G., Coppinger T. (2018). Policy determinants of physical activity across the life course: A ‘DEDIPAC’ umbrella systematic literature review. Eur. J. Public Health.

[51] Aleksovska K., Puggina A., Giraldi L., Buck C., Burns C., Cardon G., Carlin A., Chantal S., Ciarapica D., Colotto M. (2019). Biological determinants of physical activity across the life course: A “Determinants of Diet and Physical Activity” (DEDIPAC) umbrella systematic literature review. Sports Med. Open.

[52] Jaeschke L., Steinbrecher A., Luzak A., Puggina A., Aleksovska K., Buck C., Burns C., Cardon G., Carlin A., Chantal S. (2017). Socio-cultural determinants of physical activity across the life course: A ‘Determinants of Diet and Physical Activity’ (DEDIPAC) umbrella systematic literature review. Int. J. Behav. Nutr. Phys. Act..

[53] O’Donoghue G., Kennedy A., Puggina A., Aleksovska K., Buck C., Burns C., Cardon G., Carlin A., Ciarapica D., Colotto M. (2018). Socio-economic determinants of physical activity across the life course: A “DEterminants of DIet and Physical Activity” (DEDIPAC) umbrella literature review. PLoS ONE.

[54] Cortis C., Puggina A., Pesce C., Aleksovska K., Buck C., Burns C., Cardon G., Carlin A., Simon C., Ciarapica D. (2017). Psychological determinants of physical activity across the life course: A “DEterminants of DIet and Physical Activity” (DEDIPAC) umbrella systematic literature review. PLoS ONE.

[55] Condello G., Puggina A., Aleksovska K., Buck C., Burns C., Cardon G., Carlin A., Simon C., Ciarapica D., Coppinger T. (2017). Behavioral determinants of physical activity across the life course: A “DEterminants of DIet and Physical Activity” (DEDIPAC) umbrella systematic literature review. Int. J. Behav. Nutr. Phys. Act..

[56] WHO Regional Office for Europe (2016). Urban Green Spaces and Health.

[57] Molenberg F.J.M., Panter J., Burdorf A., van Lenthe F.J. (2019). A systematic review of the effect of infrastructural interventions to promote cycling: Strengthening causal inference from observational data. Int. J. Behav. Nutr. Phys. Act..

[58] Fiedler J., Eckert T., Burchartz A., Woll A., Wunsch K. (2021). Comparison of Self-Reported and Device-Based Measured Physical Activity Using Measures of Stability, Reliability, and Validity in Adults and Children. Sensors.

[59] Prince S.A., Adamo K.B., Hamel M.E., Hardt J., Connor Gorber S., Tremblay M. (2008). A comparison of direct versus self-report measures for assessing physical activity in adults: A systematic review. Int. J. Behav. Nutr. Phys. Act..

